# Dietary Supplementation with Omega-3-PUFA-Rich Fish Oil Reduces Signs of Food Allergy in Ovalbumin-Sensitized Mice

**DOI:** 10.1155/2012/236564

**Published:** 2011-11-17

**Authors:** Olívia Gonçalves de Matos, Sylvia Stella Amaral, Pedro Elias Marques Pereira da Silva, Denise Alves Perez, Débora Moreira Alvarenga, Adaliene Versiani Matos Ferreira, Jacqueline Alvarez-Leite, Gustavo Batista Menezes, Denise Carmona Cara

**Affiliations:** ^1^Departamento de Morfologia, Instituto de Ciências Biológicas, Universidade Federal de Minas Gerais, 31270-901 Belo Horizonte, MG, Brazil; ^2^Laboratório Imunofarmacologia, Departamento de Bioquímica e Imunologia, Instituto de Ciências Biológicas, Universidade Federal de Minas Gerais, 31270-901 Belo Horizonte, MG, Brazil; ^3^Departamento de Enfermagem Básica, Escola de Enfermagem, Universidade Federal de Minas Gerais, 31270-901 Belo Horizonte, MG, Brazil; ^4^Departamento de Bioquímica e Imunologia, Instituto de Ciências Biológicas, Universidade Federal de Minas Gerais, 31270-901 Belo Horizonte, MG, Brazil

## Abstract

We investigated the effect of dietary supplementation with n-3 PUFA (fish oil source) in an experimental model of food allergy. Mice were sensitized (allergic group) or not (nonallergic group) with OVA and were fed with OVA diet to induce allergy signals. Mice were fed with regular diet in which 7% of lipid content was provided by soybean (5% of n-3 PUFA) or fish (25% of n-3 PUFA) oil. Allergic group mice had increased serum levels of antiovalbumin IgE and IgG1 and changes in small intestine, characterized by an increased edema, number of rolling leukocytes in microcirculation, eosinophil infiltration, mucus production, and Paneth cell degranulation, in comparison to non-allergic group. All these inflammatory parameters were reduced in mice fed high-n-3-PUFA diet. Our data together suggest that diet supplementation with n-3 PUFA from fish oil may consist of a valid adjuvant in food allergy treatment.

## 1. Introduction

The normal immune response to dietary proteins is associated with the induction of oral tolerance, which involves a modification of the antigen in the lumen by gastrointestinal enzymes, the posterior contact with specific antigen-presenting cells with distinct activation requirements, and activation of regulatory T cells. It is well accepted that a breakdown in oral tolerance mechanism or a failure of induction of oral tolerance results in food allergy [1, 2]. 

Food allergies are disorders that affect about 20–30% of the human population in developing countries, making them some of the most common chronic diseases [3]. It is generally accepted that 6–8% of all children below 3 years of age present food allergy reactions [4, 5], specially IgE-mediated hypersensitivities [6]. Milk, eggs, peanuts, chestnuts, and shrimp are commonly related to food allergy episodes [4, 7]. In these atopic patients, continuous involuntary exposure to a food allergen may induce a mild and persistent allergic condition involving skin, gastrointestinal, and respiratory tracts disorders or trigger a multiple-organ system reaction with cardiovascular collapse [8]. Therefore, there is considerable interest in identifying interventions that are able to prevent or modify this pathological condition.

The main treatment strategy for most food allergies is based on allergen avoidance, which may present potential adverse nutritional deficiencies related to inadequate growth, neurological development, and cardiovascular health [9, 10]. Therapeutic strategies under study include oral immunotherapy [11], vaccines [12], Chinese herbal medicines [13], and dietary supplementation strategies with antioxidants [14]. Another available therapeutic option is the use of essential fatty acids for the prevention and treatment of symptoms of allergies, once the increased prevalence of allergies has been associated with modern dietary style (increased consumption of n-6 polyunsaturated fatty acids (n-6 PUFA) and decreased n-3 polyunsaturated fatty acids intake (n-3 PUFA)) [15, 16]. However, there is no clear evidence regarding modulation of immunological profile with use of n-3 PUFA during allergy. 

Based on this, in the present study we evaluated the effect of chronic intake of n-3 PUFA in a murine model of food allergy. In order to simulate this persistent food allergy situation, we used an experimental model of food allergy in which ovalbumin- (OVA-) sensitized BALB/c mice are given the antigen orally [17]. This model mimics several pathological changes that occur in patients with food allergy including increased anti-OVA IgE and IgG1 production, intestinal edema, and eosinophil infiltration in the small intestine [18].

## 2. Materials and Methods

### 2.1. Animals

Female BALB/c mice at four weeks of age were obtained from our animal facility (ICB/UFMG). All mice have received water and food *ad libitum*. The procedures were in accordance with the Ethical Principles in Animal Experimentation, adopted by the Ethics Committee in Animal Experimentation of our institution (CETEA/UFMG). 

### 2.2. Diet

**Figure 1:**
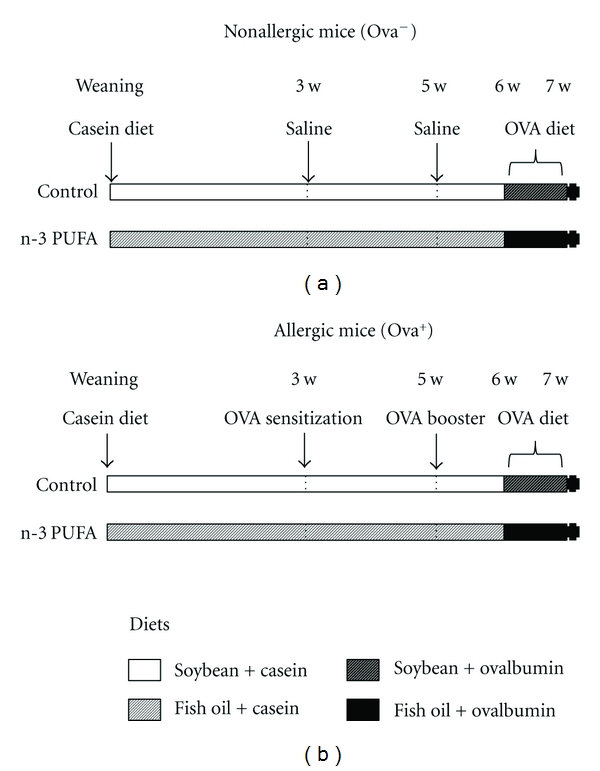
Experimental protocol. BALB/c mice were separated in 4 groups: control/nonallergic, control/allergic, n-3 PUFA/nonallergic, and n-3 PUFA/allergic. According to the diet, mice received 5% n-3 PUFA (control group) or 25% n-3 PUFA (n-3 PUFA group) as source of lipids in their diet since the beginning of the experiment (after weaning) until the end (7th week). According to the immunological procedures, mice were sensitized and received a booster (allergic group) or not (nonallergic group) with ovalbumin. Seven days after the booster, all mice received OVA diet (the diet remained with the same profile of lipids but the source of the protein was changed from casein to ovalbumin). After 7 days, the mice were sacrificed and the serum and tissues collected for analyses.

Two different diets were prepared in accordance with AIN-93 components [19], varying in fatty acid composition. Mice were fed with regular diet in which 7% of lipid content was provided by soybean (Control group: 5% of n-3 PUFA) or fish oil (n-3 PUFA group: 25% of n-3 PUFA). The diet consumption was started just after weaning in 4-week-old mice (Figure 1). The fatty acid composition of the diet is showed in Table 2. Fresh diet was given every 2 days to avoid lipid oxidation. All diets contained 14% of protein (casein before antigen challenge and ovalbumin for the antigen challenge; Tables 1 and 2). The fatty acids profile of fish oil used in this study was examined by the Chemistry Department of the Exact Sciences Institute (ICEx/UFMG) using gas chromatography. The fatty acids profile of the soybean oil used (Lisa) was obtained by Sanibal and Mancini Filho, 2004 [20].

### 2.3. Mice Sensitization and Oral Challenge

After 21 days (week 3 of experiment) of diet consumption (without OVA), allergic group (OVA**^+^**) received 0.2 mL saline (0.9%) with adjuvant (1 mg Al(OH)_3_) and 10 *μ*g OVA (five times crystallized hen's egg albumin; Sigma, St. Louis, Mo, USA). The nonallergic group (OVA**^−^**) received only saline and adjuvant. After 14 days (week 5), an immunological booster was given with 10 *μ*g OVA to allergic group. At the same time, nonallergic mice received saline. All injections were performed subcutaneously. After 7 days of booster (week 6), the two different diets remained with the same composition regarding lipids levels; however, for all groups casein was replaced by ovalbumin (lyophilized egg white-Salto's, Belo Horizonte, MG, Brazil) to induce allergic manifestations in sensitized mice during 7 days (Figure 1).

### 2.4. Serum Antibody Evaluation

After 7 days of continuum challenge with OVA diet (week 7), all mice were anesthetized with an i.p. injection of a mixture of 10 mg/kg xylazine and 200 mg/kg ketamine hydrochloride. Serum was collected for anti-OVA IgG1 and IgE analyses. ELISA for IgG1 was carried out using plates coated with OVA and 100 *μ*L of 1 : 8000 diluted mouse sera, with goat anti-mouse IgG1 (Southern Biotechnology Associates, Birmingham, Ala, USA) and rabbit anti-goat labeled with peroxidase (Southern Biotechnology Associates, Birmingham, Ala, USA). The plates were developed with o-phenylenediamine and H_2_O_2_ and were read at 492 nm on an automated ELISA reader (EL800, Bio-Tek Instruments, Inc., Winooski, Vt, USA). Anti-OVA IgE was measured by capture-ELISA using plates coated with rat anti-mouse IgE, 50 *μ*L of serum, and biotinylated OVA, as previously described [21]. The results for both antibodies are reported in arbitrary units (1000 A.U) according to the standard curve obtained with serial dilutions of pooled serum from OVA-hyperimmunized BALB/c mice.

### 2.5. Histological Analysis

After 14 days of oral challenge, the mice were sacrificed by cervical dislocation. The proximal jejunum was taken for histological analysis. It was fixed in 10% formalin in PBS, embedded in paraffin and cut into 5 *μ*m thick sections. The sections were stained with periodic acid Schiff (PAS) for mucus analysis or with hematoxylin-eosin to evaluate eosinophil infiltration. Ten fields from hematoxylin-eosin-stained sections were randomly chosen at 40x (53.333 *μ*m^2^/field) in order to count the number of eosinophils, and the data are reported as number of eosinophils/field. For mucus analysis, three sections of the jejunum stained with periodic acid Schiff were submitted to morphometric analysis using an image analysis program running on an IBM computer. Images were obtained at 40x (53.333 *μ*m^2^/field) with a JVC TK-1270/RGB microcamera and analyzed with the KS300 software built in a Kontron Eletronick/Carl Zeiss image analyzer. For the determination of goblet cell volume, all pixels with green hues were selected for the creation of a binary image and subsequent calculation of the total area, and data were reported as a percentage of mucus area/total area.

### 2.6. Intravital Microscopy of Intestine Microcirculation

To study leukocyte recruitment *in vivo*, animals were anesthetized and the abdomen was opened via a midline incision. The mice were maintained in constant temperature (34°C). A segment of small intestine was chosen and placed onto a stage, and the microcirculation was imaged using intravital microscopy by fluorescence microscopy (OLYMPUS BX41). Rhodamine 6G was used for visualization of rolling and adhered cells in intestinal microcirculation. Rolling was measured by counting the number of cells that passed for a given point during 3 minutes (cells/min) and cells that remained stopped for 30 seconds in the same point were counted as an adherent cell.

### 2.7. Statistical Analysis

The results were expressed as the mean ± SEM, as indicated in the figure legends. Significance was determined by the ANOVA-Tukey and Student *t*-tests, with *P* < 0.05 defining significance over the control group.

## 3. Results

### 3.1. Evaluation of Serum Anti-OVA IgG1 and IgE Antibodies

**Figure 2:**
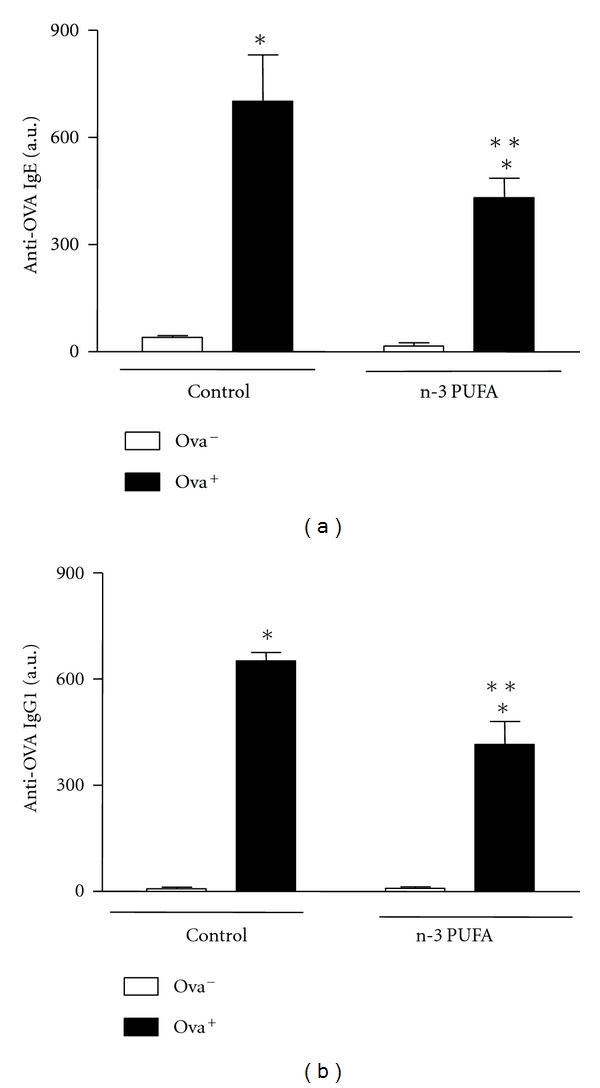
Dietary supplementation with n-3 PUFA decreases serum concentrations of anti-OVA IgE and IgG1 in BALB/c-sensitized mice. BALB/c mice received 5% n-3 PUFA (control group) or 25% n-3 PUFA (N-3 PUFA group) as source of lipids in their diet 21 days before the sensitization. BALB/c mice were sensitized (allergic, OVA^+^) or not (nonallergic, OVA^−^) with OVA. Seven days after the booster, all mice received OVA diet. After 7 days, the mice were sacrificed and the serum was collected for measurement of anti-OVA IgE and IgG1 by ELISA. Data are reported as means ± SEM for 5 animals/group. *P < 0.05 compared to nonallergic group (OVA^−^) with the same diet, and **P < 0.05 compared to allergic control group (ANOVA-Tukey).

The experimental allergy protocol induced a significant increase in serum levels of specific anti-OVA IgE and IgG1. Interestingly, n-3-PUFA-supplemented mice had significant lower levels of specific anti-OVA IgE compared to control group (Figure 2).

### 3.2. Intestinal Histology Analyses

**Figure 3:**
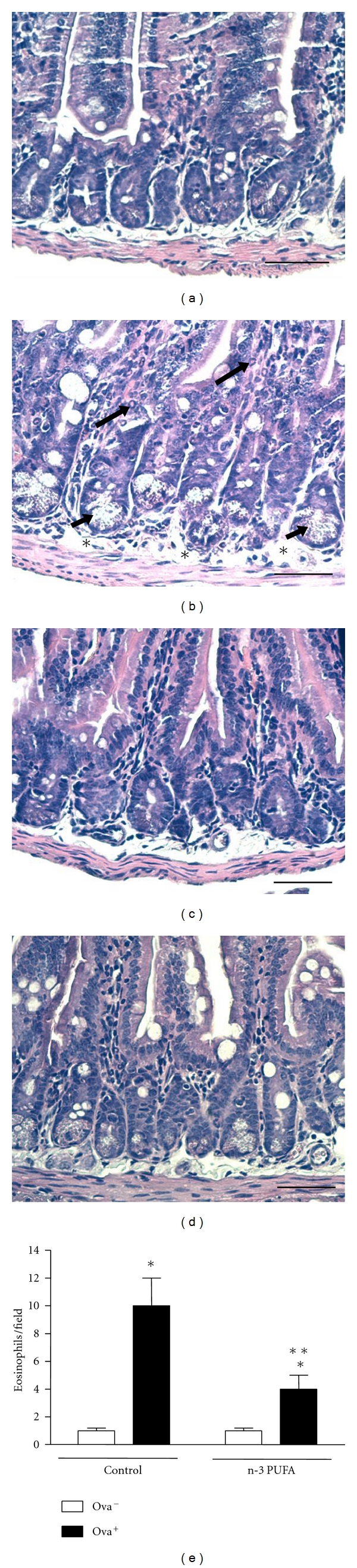
Dietary supplementation with n-3 PUFA decreases histological inflammatory parameters in jejunum of BALB/c-sensitized mice. BALB/c mice received 5% n-3 PUFA (control group) or 25% n-3 PUFA (N-3 PUFA group) as source of lipids in their diet 21 days before the sensitization. BALB/c mice were sensitized (allergic, OVA^+^) or not (nonallergic, OVA^−^) with OVA. Seven days after the booster, all mice received OVA diet. After 7 days, the mice were sacrificed and the intestine was taken for histology analyses. (a) Nonallergic control; (b) allergic control; (c) nonallergic n-3 PUFA; (d) allergic n-3 PUFA. Bar = 50 *μ*m. In (b), long arrows show eosinophils, short arrows show Paneth cells, and asterisks show submucosal edema. In (e), data are reported as means ± SEM of number of eosinophils for 5 animals in each group. *P < 0.05 compared to nonallergic group (OVA^−^) with the same diet, and **P < 0.05 compared to allergic control group (ANOVA-Tukey).

The ingestion of OVA diet induced submucosal edema and increased degranulation of Paneth cells and inflammatory cell infiltration in mucosa. There was 10-fold increase in the number of eosinophils in control allergic group compared to nonallergic mice. Interestingly, edema and eosinophil infiltration were significantly reduced in mice fed increased n-3 PUFA diet. Also, Paneth cells from n-3 PUFA-supplemented mice displayed a regular profile of degranulation, similar to controls (Figure 3).

### 3.3. Evaluation of Intestinal Mucus by Goblet Cells

**Figure 4:**
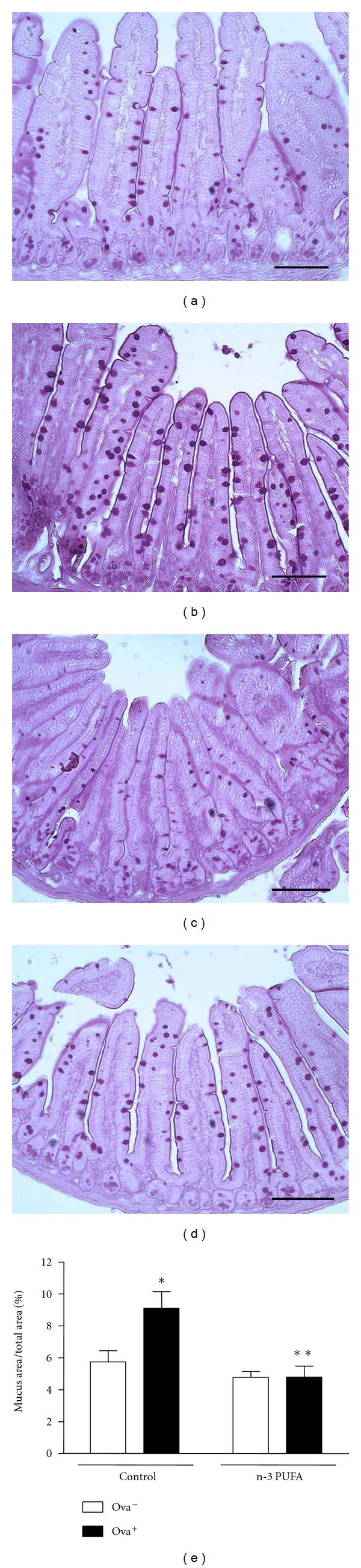
Dietary supplementation with n-3 PUFA decreases mucus production in small intestine of BALB/c-sensitized mice. BALB/c mice received 5% n-3 PUFA (control group) or 25% n-3 PUFA (N-3 PUFA group) as source of lipids in their diet 21 days before the sensitization. BALB/c mice were sensitized (allergic, OVA^+^) or not (nonallergic, OVA^−^) with OVA. Seven days after the booster, all mice received OVA diet. After 7 days, the mice were sacrificed and the intestine was taken for histology analyses. (a) Nonallergic control; (b) allergic control; (c) nonallergic n-3 PUFA; (d) allergic n-3 PUFA. Bar = 50 *μ*m. In (e), data are reported as means ± SEM of mucus production for 5 mice in each group. *P < 0.05 compared to nonallergic group (OVA^−^) with the same diet, and **P < 0.05 compared to allergic control group (ANOVA-Tukey).

Continuous exposure to the antigen induced a significant increase in mucus production by goblet cells in the small intestine of sensitized wild-type BALB/c mice when compared to nonsensitized animals. On the other hand, antigen ingestion induced no increase in mucus secretion in the small intestine of mice fed with n-3 PUFA diet (Figure 4).

### 3.4. Intravital Microscopy of Intestine

**Figure 5:**
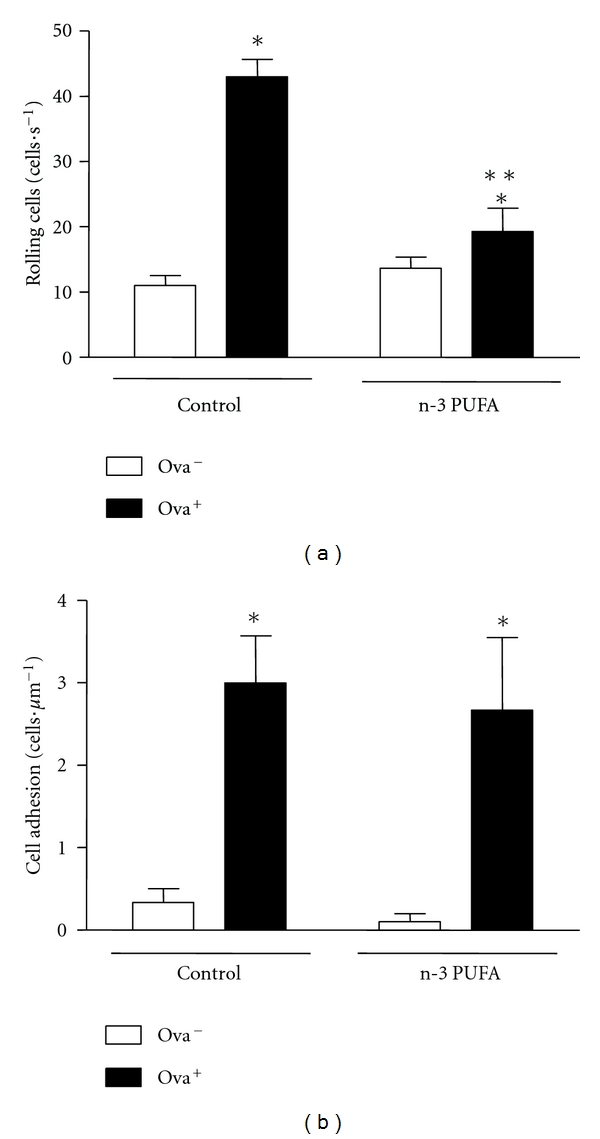
Dietary supplementation with n-3 PUFA decreases leukocyte rolling in small intestine of BALB/c-sensitized mice. BALB/c mice received 5% n-3 PUFA (control group) or 25% n-3 PUFA (n-3 PUFA group) as source of lipids in their diet 21 days before the sensitization. BALB/c mice were sensitized (allergic, OVA^+^) or not (nonallergic, OVA^−^) with OVA. Seven days after the booster, all mice received OVA diet. After seven days of ingestion of the ovalbumin- (OVA-) containing diet, the leukocyte rolling (a) and adhesion (b) to the microvasculature of intestine were assayed by intravital microscopy. Data are reported as means ± SEM for 3 mice in each group. *P < 0.05 compared to control group (ANOVA-Tukey).

Leukocyte recruitment is a hallmark feature of the inflammatory response, and it involves a sequential series of molecular interactions between the leukocyte and endothelial cells [22]. Once we have detected a reduced eosinophil infiltration in n3-PUFA group, we decided to investigate, by using intravital microscopy, in which step of leukocyte recruitment this diet was interfering. Allergic group had an increase in rolling and adhered leukocyte number in intestinal microvasculature after OVA diet. However, mice fed n-3 PUFA diet displayed a reduction in the total number of rolling leukocytes (Figure 5(a)) with no differences in the number of adherent cells (Figure 5(b)).

## 4. Discussion

Immunoglobulin-E-dependent food allergy typically affects the gastrointestinal tract with different degrees of eosinophilic inflammation and edema [8]. Food allergy treatment is mostly based on pharmacological approach (mainly antihistaminic and corticoids) and food antigen avoidance, being the last one the only efficacious alternative in several refractory patients. In this sense, supplements with ability to decrease or avoid allergic reactions against food contents may be promising. In the food allergy model used in this study, when ovalbumin-sensitized mice were given OVA (antigen) in the diet, several signs of food allergy were observed, including increased serum antiovalbumin IgG1 and IgE and marked histological findings of intestinal inflammation (mucus hypersecretion, eosinophil infiltration, Paneth cell degranulation, and edema) [17]. In the present work, we have shown that OVA allergic mice had a less severe allergic response when the polyunsaturated fatty acid omega 3 was increased in the diet. We provided evidence that reduced OVA-specific IgE production by n-3-PUFA-supplemented diet led to reduced eosinophil infiltration into gut mucosa, with mild intestinal inflammatory response in mice. These data together suggest that food supplementation with n-3 PUFA may consist of a promising venue to treat food-associated allergic disorders despite food avoidance. 

The known mechanism involved in IgE-dependent food allergy is attributed to the generation of Th2 cells that produce IL-4 with further generation of IgE and IgG1 antibodies [8]. These immunoglobulins bound in mast cells via its high affinity receptor (Fc*ε*RI), leading to release of a large number of proinflammatory mediators and proteases into adjacent tissues [23]. Also, these activated mast cells produce Th2-type cytokines, including IL3, IL-5, and IL-13, leading to the accumulation of eosinophils which promote the expansion of Th2 cells in inflamed tissues and release of proinflammatory mediators with upregulation of adhesion systems, modulation of cellular trafficking, activation and regulation of vascular permeability and mucus secretion, and tissue damage [24]. The mucosal immune system accounts for a number of mechanisms to avoid an uncontrolled immune response against food antigens, including the presence of regulatory T cells in the lymphoid tissue of the gut [25] and mucus (a physical barrier against antigens) [26]. Consistent with this, a reactive increase of mucus production is expected during food allergy model, which is reduced in the absence of IL-4 and IgE [27]. Additionally, gut mucosa cells play a key role in the digestive tract homeostasis. Paneth cells are secretory cells in the epithelium of the small intestine, which reside in small clusters at the base of crypts of Lieberkühn, and they are the main source or antimicrobial peptides in gut [28]. These peptides are described to act protecting gut mucosa against enteric bacterial pathogens, participating as a key homeostatic role in establishing and maintaining the intestinal microbiota [29]. In our study, histological evaluation showed that during food allergy a degranulation profile is observed in Paneth cells. Although there is no known direct correlation between Paneth cells and food allergy, we suggest that these cells may be involved in allergy-induced gut mucosa inflammation.

 The polyunsaturated acids (including n-3 and n-6 PUFA) are precursors of several eicosanoids, such as prostaglandins (PG), leukotrienes, thromboxanes, and hydroxyeicosatetraenoic acids. Consumption of n-6 PUFA leads to the formation of specific eicosanoids with a proinflammatory profile, which has an essential role in allergic inflammation. Thereby, linoleic acid (LA; C18 : 2 n-6), one of the major dietary n-6 PUFA, is converted to arachidonic acid (AA; C20 : 4 n-6) and is incorporated into membrane phospholipids [30]. During metabolism, AA is released by phospholipases and metabolized to PGE2, driving a Th2 subset response. In this sense, imbalanced dietary intake of n-6 PUFA may increase the predisposition to atopic disorders with IL-4 and consequent IgE production. In sharp contrast, an increased n-3 PUFA ingestion will lead to a metabolic competition with n-6 PUFA metabolism, culminating in decreased synthesis of PGE2, decreased IL-4 and IgE, as seen in our model [27]. Additionally, n-3 PUFA supplementation in allergic subsets may be beneficial, since a less inflammatory environment may be achieved during fatty acid metabolism [31].

In fact, n-3 PUFA supplementation leads to a less severe inflammation in gut mucosa from allergic mice and decreased production of IgE. This statement may be strengthened by three major findings. First, lower levels of IgE were observed in supplemented mice. Mast cells, which are extremely activated by IgE, can release several mediators upon IgE activation, IL-5 and eotaxin being potent chemoattractants to eosinophils. This may explain the marked reduction in eosinophils observed in n-3-PUFA-supplemented mice. In fact, a previous report has shown that both DHA and EPA are able to decrease the chemotactic and chemokinetic responses of eosinophils in a dose-dependent fashion [31]. Second, we observed less mucus production and Paneth cells degranulation in n-3-PUFA-supplemented mice, which is a clear histological indication of a mild gut inflammatory response [27]. And finally, intravital microscopy of intestinal microvasculature revealed that food enriched with n-3 PUFA by fish oil led to a reduction in the total number of rolling leukocytes, as an indication of reduced endothelial-leukocyte interaction. Several reports have shown that consumption of n-3 PUFA in fish oil may reduce the inflammatory response in several chronic inflammatory diseases characterized by leukocyte accumulation such as atherosclerosis, asthma, systemic lupus erythematosus, inflammatory bowel disease, and rheumatoid arthritis [32–35]. In a previous report it was shown that oxidized EPA is a potent inhibitor of leukocyte interaction with the endothelium. The proposed mechanism responsible for this effect seems to be the activation of nuclear receptor peroxisome proliferator-activated receptor *α* (PPAR*α*) and subsequent downregulation of leukocyte adhesion receptor expression [36]. 

Our results are consistent with previous works such as that performed by Watanabe et al. which showed that the IgE antibody response against egg albumin was significantly lower in the mice fed with safflower seed oil [37]. Also, Yamashiro et al. have shown that the mucosal damage induced by intestinal hypersensitivity reactions to ovalbumin is regulated by omega-3-fatty-acid enriched diet [38]. On the other hand, depending on the model or on the feeding design, the results can be controversial. For example, Johansson et al. have shown that, during the airway hypersensitivity (Th2), mice fed with fish oil produced high levels of OVA-specific IgE and had slightly high eosinophil infiltration into the lungs. Contrastingly, chronic n-3 PUFA consumption (as shown in our study) or lipid-based allergy prevention performed since in the uterus, via maternal diet [39, 40], provide evidence of benefic immunological modulation and less inflammatory tissue damage. Further basic investigation may provide guidelines for new trials, since meta-analysis studies have not confirmed the beneficial role of n-3 or n-6 PUFA supplementation as a strategy for the primary prevention of food allergy [41]. 

In conclusion, we have shown that diet supplementation with n-3 PUFA from fish oil led to a reduction of gut inflammatory response against food antigen, which suggests that n-3 PUFA may modulate the allergic immune response.

## Figures and Tables

**Table 1:** Mouse chow ingredients (based on AIN-93G diet).

Ingredient	g/kg diet
Protein source	
Casein (day 0–21)	200.000
or	
Ovalbumin (day 21—end)	200.000
Lipid source	
Soybean oil	70.000
or	
Fish oil	70.000
Cornstarch	529.500
Sucrose	100.000
Fiber (cellulose)	50.000
Mineral mix (AIN-93G-MX)	35.000
Vitamin mix (AIN-93-VX)	10.000
L-cystine	3.000
Choline bitartrate (41.1% choline)	2.500
Tert-butylhydroquinone	0.014

**Table 2:** Fatty acid composition of mouse diets.

Fatty acid	Fish oil (%)	Soy oil (Lisa) (%)
Sum of SFA	30.8	15.24
Sum of MUFA	29	22.69
C18 : 2 n-6c (LA)	1.3	55.83
C18 : 3n6 (GLA)	0.2	
C20 : 4 n-6c (AA)	0.0	
Sum of n-6PUFA	1.5	55.83
C18 : 3n3 (ALA)	0.6	4.79
C20 : 3n3	1.3	
C20 : 5 n-3 (EPA)	15.9	0.0
C22 : 6 n-3 (DHA)	7.9	0.0
Sum of n-3 PUFA	25.7	4.79
SD	13	0.07

Total	100	100

SFA: saturated acid; MUFA: monounsaturated acid; LA: linoleic acid; GLA: *γ*-linolenic acid; AA: arachidonic acid; ALA: *α*-linolenic acid; DHA: docosahexaenoic acid; EPA: eicosapentaenoic acid; SD.
